# 

**Published:** 1891-12-05

**Authors:** 


**Notices of Births, Deaths, and Marriages**, are inserted in this column at a charge of 2s. 6d. for an announcement not exceeding four lines.

### NOTICE TO CORRESPONDENTS.

All MS., Letters, Books for Review, and other matters intended for the Editor should be addressed The Editor, The Lodge, Porchester Square, London, W.

The Editor cannot undertake to return rejected M.S., even when accompanied by stamped directed envelope.

### Notice to Advertisers and Subscribers.

All Advertisements, Orders for Copies of The Hospital, and Business Communications should be addressed to The Publisher, *not to the Editor*, The Hospital, 140, Strand, W.C.

The Cost of Subscription, post free, is as follows:—Per week, 1½d.; per quarter, 1s. 8d.; per half-year, 3s. 3d.; per annum, 6s. 6d. Foreign:—Per annum, 8s. 8d.; payable, in all cases, in advance.

**"Burdett's Hospital Annual," 1891—1892.**

Third year of publication. 600 pp. Boards, gilt lettering. A most complete guide to British and Colonial Hospitals, Dispensaries, Nursing and Convalescent Institutions, and Asylums. Price 3s. 6d. Published by The Hospital (Limited), 140, Strand, W.C.

**NOTICE.—The Index for Vol. X. (ending September, 1891), is on sale at the Office of this Journal, price 2½d., post free.**

### GENERAL CHARITIES.

[This Column is devoted to an account of work of charitable institutions other than Medical Charities. The Editor will be glad to receive reports or news of work at 140, Strand. *Philanthropy* should be plainly written on the envelope.]

Mr. C. B. Stuart-Wortley, M.P., Under Secretary of State for the Home Department, has promised to preside at a supper to be given by the **ST. GILES' CHRISTIAN MISSION**, at the Chapel, Little Wild Street, Drury Lane. It has recently been the fashion to ask annually as guests on this occasion the criminal classes, who are generously invited both to supper and reform. The former invitation no doubt is accepted with alacrity, the latter we fear with reluctance.

Lord Robartes and the Hon. Richard Strutt have been elected to fill the vacancies upon the Court of the **CORPORATION OF THE SONS OF THE CLERGY**, caused by the deaths of Earl Beauchamp and Mr. Frederick Calvert. At the recent sitting of the Court, which granted the sum of £1,530 in response to 131 applications, aid was given towards the expense of emigrating, preparing for various professions, among them that of an electrical engineer, and entering upon situations, besides the usual help towards the education of clergymen's sons. The number of applications was quite unusual.

A meeting to forward the work of the musical branch of the **KYRLE SOCIETY**, was held last week under the presidency of Lord Monkswell at 24, Park Lane. As Lord Monkswell remarked many people are cognisant of this work only in a vague sort of way, though its operations extend far from the centre of London. That the society has aroused and stimulated a taste for high-class music amongst the poor cannot be doubted. Some years ago it was the fashion in certain quarters to throw cold water on these efforts. The English are not a musical people, it is alleged; therefore why waste money in trying to educate a people who are deaf, charm you never so sweetly. Apropos of this view of the question, Mr. Sala told those present how in his youth his mother announced a performance of the *Stabat Mater* at Richmond, but the project was preached down by the local clergy and had to be abandoned, and mentioned as a significant circumstance the fact that the same work was performed only a few days ago in a chapel of the Congregational Union. It appears from the report of the Sub-committee that 22 oratorio performances had been given by the society at London churches during the past year, and, in addition to the music in public gardens, 18 concerts and entertainments had been given. Increased support is now needed if this work is to be continued, and for this support the President and others appealed. Another object of the society, which was not mentioned at this meeting, but which is, we think, equally worthy of support, is to brighten the existence in workhouses, infirmaries, and other such institutions, by the loan of books. Presents of wholesome literature, whether light or serious, are always welcome at 49, Manchester Street, and those who have books which they do not care to keep in their libraries might thus place them within the reach of others who can appreciate them although they cannot afford to buy them.

In the middle of a seething population in the heart of the East-end of London is an institution, and it is the only one of its kind, called the **EAST-END MOTHERS' HOME**. Its aim is to provide a homely shelter, with privacy, good attendance, and skilled nursing, for those women of the working classes to whom the birth of a child means, as the Committee very truly point out, "a season of extra expense, anxious care, privation, discomfort, illness, and sometimes even death." This Home, which is situated at 395, Commercial Road, E., and has not more than an income of £1,000 a-year from all sources, and which has to serve the needs of a very poor and numerous population, has many interesting features. It not only does an infinite amount of direct good at a time of greatest necessity to the women themselves, but indirectly many husbands who see the cleanliness and cheerful contentedness of their wives in the Home are led to institute comparisons with their own home which can have none other but good effect. Though patients are expected to bring vouchers of respectability they are received gratis, and each woman has a small plainly furnished room to herself. Thus, and through the efforts of the Lady Superintendent to cultivate a home feeling, good influences surround the patient which are still powerful when she returns to her home. Owing to the bounty of a subscriber an out-patient department has been opened, which will no doubt prove an additional boon, but the home itself only contains ten beds. The building, which is on a freehold site, is capable of extension, but most unfortunately funds are not only lacking for this purpose but the Home is in urgent need of prompt pecuniary assistance. Only those who know the crowded dwellings which exist in parts of our great towns can describe, if words can be found adequate to the task, the trials which many women have to go through when the peril of child-birth is added to their burdens. Their more prosperous sisters have a noble opportunity at this season of charity and good will of doing a good deed by bringing to these daughters of toil and sorrow the skilled attendance, good food, and rest in their hour of need. Let them, therefore, and all who are blessed with the good things of this world, send donations to the Treasurer, Mr. Percy Wigram, 12A, Savill Row, W. It would be little short of a national scandal if such an institution, situated where in all London it is most needed, were allowed to languish for want of funds.

### SCRAPS AND GLEANINGS.

HALIFAX is seriously entertaining the project of a new hospital. A very necessary undertaking.

AN amalgamated medical teaching scheme is suggested at the Queen's and General Hospitals, Birmingham.

BY the death of Mr. R. A. Newbon, the Great Northern Central Hospital benefits to the extent of £15,000.

SIX hundred and thiry-one pounds has already been subscribed for the proposed Cottage Hospital for Cupar, N.B.

THE scheme for a Cottage Hospital at Chipping Norton has been abandoned in favour of a Nursing Association.

THE Marquis of Salisbury has become a life member of the National Society for the Prevention of Cruelty to Children.

THE Registrar-General's returns for the week ending November 21st, showed that there were seven deaths from influenza.

LORD GEORGE HAMILTON has consented to present the prizes at the Princess Helena College on December 8th, at three o'clock.

THE anniversary service in aid of the funds of the Liverpool and Southern Hospital has produced £123 6s. for the institution.

THE Worshipful Company of Skinners has made a donation of 100 guineas to the London Young Women's Christian Association Extension Fund.

THE Queen has forwarded a portrait of herself to be hung in the day room of the Morley House Convalescent Home for Working Men at Dover.

WE cannot quite understand the report of the Faversham Cottage Hospital, appearing in a local paper. It is stated that 55 patients occupied 1,973 beds.

THE Prince of Wales, under warrant dated November 9th, 1891, has specially appointed Messrs. J. Brinsmead and Sons pianoforte manufacturers to H.R.H.

TYPHOID fever has broken out in Mildysart, and already two deaths have occurred. A public meeting is to be held for the purpose of condemning the authorities for bad sanitary arrangements.

MANY people are inclined to think that circular appeals are a dead letter. The Denbighshire Infirmary fortunately has not found this to be the case. Donations after an appeal amounted to £400.

ATTENTION has been called to the financial condition of the General Hospital at Nottingham. The hospital is in debt to the Bank to the extent of nearly £3,000; 50 beds will consequently have to be closed.

A NOTTINGHAM resident suggests that if workmen who make more than 25s. a-week would forfeit 1d. a-week of their earnings to their employer this would serve as an insurance fund against poverty and disease.

A VERY interesting little history of "The Hospitals of Ancient Stirling" appears in the *Stirling Journal* for the 20th of this month; it is well worth the attention of our readers who are interested in the subject.

LORD SHEFFIELD, one of the Lords-in-Waiting and Superintendent of the Stables in the Prince of Wales's Household, has been suffering from an attack of influenza in Belfast, but according to the latest accounts he is now better.

THE majority of the cases of fever on board the Boadicea were contracted at Malta, where fever may be regarded as epidemic. Very many of them were of a mild nature. No deaths have been reported up to the present time.

IT has been decided by the Leeds Corporation to erect another hospital owing to the continuous spread of scarlet fever, the existing accommodation being inadequate for the fresh cases which have to be dealt with. The epidemic of small-pox is fast disappearing.

A WOMAN in Devonshire has died of the effects of a dose of strychnine, "dispensed" to her by a dentist's assistant instead of morphia. The verdict was "Death by Misadventure," and the chemist was merely "censured"—*pour encourager les autres*, we suppose.

WE regret to see that the Derbyshire Children's Hospital, an admirably managed institution, is still in want of funds. It is to be hoped that the ladies of Derbyshire will follow the suggestion made, and endeavour to make collections in aid of the institution.

THE attention of the Sanitary Authorities should be earnestly directed to the considerable amount of diphtheria prevalent in the metropolis. In some districts, notably Paddington, a number of severe cases have occurred, several terminating fatally.

HER Majesty's ships, Marathon and Redbreast, reported from Port Said, were not put in quarantine on account of cholera on board. During the stay of the vessels at Bombay cases of cholera were present, and there is evidence that the disease was contracted by men on leave there and so conveyed to the ships.

A PORTSMOUTH telegram says letters received report a serious condition of things on board the *Boadicea*, the new flagship on the East Indies station. She left Port Said 114 men short of her complement, 30 were sent to the hospital at Malta, and 30 at Port Said. There are several more cases of fever on board.

THE Society for Promoting Christian Knowledge will again this year send parcels of picture cards and small books to various institutions all over the country, containing a large number of juvenile patients, for distribution among these little sufferers on Christmas Day. Last year 5,000 little ones in 79 hospitals, &c., received a gift from the society.

A SERIOUS outbreak of diphtheria has, it is stated, occurred at Bishopstoke, Hants. During the past week several children have died, and others are in a very precarious state. It has been necessary to close the local Board schools, which are fitted up as a temporary hospital, under the care of a trained nurse. No cause is at present assigned for the outbreak.

THE last accident that has happened to a wild-beast tamer in France—whose eyes a bear scratched out—seems to have suggested a novel safeguard, to a chemist, who suggests that an attendant should always be in readiness, with a syringe of caustic ammonia, which he can squirt into the face of any animal who turns disagreeable. The beast sneezes and the man is safe.

### THE STUDENT OF MEDICINE.

[All communications for this Supplement should be addressed to The Editor of The Hospital, at 140, Strand, with the words, "Students' Column," written in the left hand top corner of the envelope.]

### VACANCIES.

The following vacancies are announced this week, the amount of salary in each case being given after the name of the institution:

### Resident Medical Officers.

City of London Hospital for Diseases of the Chest, House Physician—Board, &c.

Dudley Dispensary, Resident Medical Officer—Salary £130.

North-Eastern Hospital for Children, Junior House Surgeon—Salary £60.

Northern Infirmary, Inverness, House Surgeon and Apothecary—Salary £100, with board, &c.

North Staffordshire Infirmary and Eye Hospital, Hartshill, Stoke-upon-Trent, House Physician—Salary £100; also Assistant House Surgeon—Board, &c.

Royal Albert Hospital, Devonport, Resident Medical Officer—Salary £100, with board, &c.

Sunderland Infirmary, House Surgeon—Salary £80, with board, &c.

West London Hospital, House Physician, also House Surgeon—Board, &c.

Wolverhampton Eye Infirmary, House Surgeon—Salary £60, with board, &c.

York County Hospital, Senior House Surgeon—Salary £100, with board, &c.

### Non-Resident Medical Officers.

City of London Hospital for Diseases of the Chest, Pathologist—Salary £105.

Dental Hospital of London, Leicester Square, Dental Surgeon, and Assistant Dental Surgeon, and also Anæsthetist.

Newcastle-on-Tyne Dispensary, Visiting Medical Assistant—Salary £120.

Royal Infirmary, Bristol, Honorary Obstetric Physician.

Western General Dispensary, Marylebone Road—Honorary Surgeon.

### SCHOLARSHIPS AND PRIZES.

### National Dental College, Great Portland Street.

Honours List, 1890-91.—Bronze Medals: Dental Anatomy, Mr. Moore; Dental Surgery, Mr. Bascombe; Dental Mechanics, Mr. Moore; Metallurgy, Mr. Bascombe; Operative Dental Surgery, Mr. Moore; Dental Materia Medica, Mr. Moore; The "Rymer" Gold Medal for General Proficiency, Mr. Moore; the "Ash" Prize of Three Guineas for the Thesis on Hæmorrhage after Tooth Extraction, Mr. Arnold Prager; Entrance Exhibition of the value of £15, Mr. M'Farline; Students' Society Prize, Mr. Keele; Mechanical Work, Mr. Keele. Certificates.—Dental Anatomy, Mr. Outts; Dental Surgery, Mr. Burbery Rowe; Dental Mechanics, Mr. Prager; Metallurgy, Messrs. Johnson and Keele; Operative Dental Surgery, Mr. Bascombe; Dental Materia Medica, Mr. Prager; Element of Histology, Mr. Bascombe.

### University of Cambridge.

The Coutts Trotter Studentship, of the annual value of £250 for the promotion of original research in Natural Science, more especially Physiology and Experimental Physics, open to graduates of Trinity College, not being Fellows, who are not of more than seven years' standing from the time of commencing residence, has been awarded to John Walton Capstick, B.A., scholar of the College.

### EVENTS FOR THE MONTH OF DECEMBER.

### Medical Societies.

St. Mary's Hospital Medical Society.—December 16th: Some Cases of Histeria, Dr. M. Handfield Jones; clinical cases, Mr. T. H. R. Crowle.

Queen's College Medical Society.—December 16th: General meetings.

Westminster Hospital Medical School.—December 10th: Meeting at eight p.m.

### Football.

St. Mary's Hospital.—First fifteen, December 5th, v. Windsor, at Windsor; 19th, v. Middlesex Wanderers (A), at home. Second fifteen, November 5th, v. University College Hospital (second), at home; 12th, v. Eltham House, at Raynes Park.

St. Thomas's Hospital.—First fifteen: December 5th, v. Clapham Rovers, at Wandsworth; December 12th, v. Croydon, at Croydon; December 19th, North v. South, at Newcastle. Second fifteen: December 5th, v. Marlboro' Nomads (second), at Surbiton; December 12th, v. Wasps (second), at Lambeth; December 19th, v. Dulwich College (second), at Dulwich.

University College and Hospital.—Hospital Team: December 5th, v. Beaumont College, at Old Windsor; December 12th, v. Ealing Club, at Ealing; December 19th, v. Royal Veterinary College, away. College Team: December 5th, v. St. George's Hospital, away.

Westminster Hospital.—December 5th, v. Clapham Park, at Hyde Farm, Balham; December 12th, Royal College of Science, at Raynes Park.

Owens College.—First Team: December 5th, v. Yorkshire College, away; December 12th, v. Stockport, at home; December 19th, v. Ratcliffe, at home; December 26th, v. Blackley, at home. A Team: December 5th, v. Manchester, at home; December 12th, v. Stockport, away; December 19th, v. Bradford Old Boys, away. B Team: December 5th, v. Broughton Park (A), away; December 19th, v. Liverpool Old Boys, at home.

Yorkshire College.—December 5th, v. Owens College, at home; December 5th, v. St. Peter's, York, away; December 12th, v. Pateley Bridge, at home; December 12th, v. Gildersome, away.

